# Effects of Conjugation with Basil Seed Gum on Physicochemical, Functional, Foaming, and Emulsifying Properties of Albumin, Whey Protein Isolate and Soy Protein Isolate

**DOI:** 10.3390/foods14030390

**Published:** 2025-01-24

**Authors:** Hadi Hashemi, Mohammad Hadi Eskandari, Mohammadreza Khalesi, Mohammad-Taghi Golmakani, Mehrdad Niakousari, Seyed Mohammad Hashem Hosseini

**Affiliations:** 1 Department of Food Science and Technology, School of Agriculture, Shiraz University, Shiraz 71441-13131, Iran; hadihashemigahruie@shirazu.ac.ir (H.H.); eskandar@shirazu.ac.ir (M.H.E.); golmakani@shirazu.ac.ir (M.-T.G.); niakosar@shirazu.ac.ir (M.N.); 2 Department of Biological Sciences, University of Limerick, V94 T9PX Limerick, Ireland; mohammadreza.khalesi@ul.ie

**Keywords:** albumin, basil seed gum, conjugation, soy protein isolate, whey protein isolate

## Abstract

Protein conjugation with the Maillard reaction has received considerable attention in the past decades in terms of improving functional properties. This study evaluated the changes in the techno-functional properties of whey protein isolate (WPI), soy protein isolate (SPI), and albumin (Alb) after conjugation with basil seed gum (BSG). The conjugates were developed via the Maillard reaction. Various analyses including FT-IR, XRD, SEM, SDS-PAGE, DSC, RVA, rheology, zeta potential, emulsion, and foaming ability were used for evaluating conjugation products. Conjugation between proteins (WPI, SPI, Alb) and BSG was validated by FT-IR spectroscopy. XRD results revealed a decrease in the peak of BSG after conjugation with proteins. SDS-PAGE demonstrated the conjugation of WPI, SPI, and Alb with BSG. DSC results showed that conjugation with BSG reduced the *T*_g_ of WPI, SPI, and Alb from 210.21, 207.21, and 210.90 °C to 190.30, 192.91, and 196.66 °C, respectively. The emulsion activity and emulsion stability of protein/BSG conjugates were increased significantly. The droplet size of emulsion samples ranged from 112.1 to 239.3 nm on day 3. Nanoemulsions stabilized by Alb/BSG conjugate had the smallest droplet sizes (112.1 and 143.3 nm after 3 and 17 days, respectively). The foaming capacity of WPI (78.57%), SPI (61.91%), and Alb (71.43%) in their mixtures with BSG increased to 107.14%, 85.71%, and 85.71%, respectively, after making conjugates with BSG. The foam stability of WPI (39.34%), SPI (61.57%), and Alb (53.37%) in their mixtures with BSG (non-conjugated condition) increased to 77.86%, 77.91%, and 72.32%, respectively, after formation of conjugates with BSG. Conjugation of BSG to proteins can improve the BSG applications as a multifunctional stabilizer in pharmaceutical and food industries.

## 1. Introduction

*Ocimum basilicum*, from the Laminaceae family, is grown in different locations, mainly in France, Italy, Egypt, Bulgaria, South Africa, Hungary, and North America [1]. Because of its pleasant and specific flavor characteristics, basil is usually used for food production. Also, basil seed is popular due to many health benefits such as relieving ulcer, cough, diarrhea, kidney disorder, indigestion, and sore throat. Therefore, it is a popular ingredient in traditional medicine [2]. The seeds’ mucilage, isolated by basil seed hydration in water, contains high amount of polysaccharides [3]. Basil seed gum (BSG) has the potential to create functional characteristics including emulsion and foam stabilization, thickening, mouthfeel improvements, and edible coating [4,5].

Improving the functional characteristics of proteins is important for the producers of food, cosmetic, and pharmaceutical products. A lot of modifications including physical, chemical, or enzymatic techniques have been applied to increase proteins’ functionality [6,7,8,9,10,11,12]. Chemical modifications due to the formation of detrimental products or potential health hazards have not been extensively used for food production. Therefore, a different strategy is needed to increase the proteins’ functional properties in the food industry.

Today, research on the polysaccharide–protein conjugation reaction, based on the Maillard-type reactions, which includes the reducing end carbonyl groups of polysaccharides and the protein’s free amino groups, have been increased. This chemical modification can improve the product’s functional properties [13,14,15]. As an example, the emulsifying properties of proteins are significantly improved by the Maillard reaction [16]. The main aspect of the prepared protein/polysaccharide conjugates is the higher foaming and emulsifying capability, which is practical for food industry [16,17]. Also, conjugation can improve the solubility of proteins, and the antioxidant and antibacterial properties of polysaccharides and proteins [11,18]. The allergenicity of some proteins is reduced by its conjugation with polysaccharides [19,20]. Furthermore, the comprehensive review by Urango et al. [21] on Maillard conjugates in health showed that the products of the Maillard reaction can modulate the intestinal microbiota composition in adolescents during in vivo testing. It can also increase immunomodulatory effects, improve immunity, increase the production of various immune cells, improve the bioaccessibility of total carotenoids, increase anti-inflammatory effects, and inhibit cell proliferation activity in human breast cancer cells.

Research on the application of protein/polysaccharides conjugates as emulsifying and foaming agents has increased. Improvement in the foam and emulsion properties by Maillard conjugates were reported in several studies in 2024 by Zhang et al. [22] on the SPI hydrolysate-various polysaccharides (e.g., gum Arabic, sodium alginate, xanthan gum, and soybean polysaccharides) conjugates, Tao et al. [23] on the SPI/soybean peptide and ginseng polysaccharide conjugates, and Amiratashani et al. [24] on the grass pea protein and xanthan gum conjugates.

This technique uses a naturally occurring reaction without the need of chemical solvents. So, it can be considered an important method for industrial application. To our knowledge, there is no report on the conjugation of whey protein isolate (WPI), soy protein isolate (SPI), and albumin (Alb) with BSG. Therefore, the aim of this study was to evaluate the changes in the physicochemical, functional, thermal, foaming, and emulsifying properties of WPI, SPI, or Alb after fabricating conjugates with BSG using the dry method under controlled temperature and relative humidity conditions.

## 2. Materials and Methods

### 2.1. Materials

BSG (moisture (5.74%), protein (1.28%), and carbohydrate (84.74%)) were obtained from Reyhan Gum Parsian (Tehran, Iran). WPI (protein > 90%), SPI (protein > 92%), and Alb (protein > 98%) were obtained from Davisco Foods International Inc. (Eden Prairie, MN, USA), G max Chemicals Co., Ltd. (Shandong, China), and Sigma Chemical Co. (St. Louis, MO, USA), respectively. Canola oil was purchased from Narges Oil Co. (Shiraz, Iran). Other reagents and materials were of analytical grade.

### 2.2. Preparation of BSG Conjugates

Proteins (100 mg) and BSG (100 mg) were mixed and dispersed in 10 mL sodium phosphate buffer (0.05 M, pH = 8.5 ± 0.1). After overnight hydration, the dispersions were lyophilized and milled into a fine powder. Powdered samples were kept in sealed glass desiccators containing saturated KBr solution (RH ~ 79%) at the bottom. Desiccators were kept in an oven at 60 °C. The conjugates (BSG/WPI conjugate, BSG/SPI conjugate, and BSG/Alb conjugate) were harvested after 10 days and evaluated [25]. Control samples (BSG/WPI mixture, BSG/SPI mixture, BSG/Alb mixture) containing non-conjugated mixture of BSG and three different types of proteins were also investigated.

### 2.3. FT-IR Spectroscopy

An FT-IR spectrometer (Avatar 370, Thermo Nicolet Corp., Madison, WI, USA) was used to study the functional groups and structural characteristics of proteins/BSG mixtures or conjugates. All samples were mixed with KBr to prepare pellets. The spectra were recorded from 4000 to 400 cm^−1^ [24].

### 2.4. Microstructure (SEM)

The surface microstructure of mixtures and conjugates were studied using a scanning electron microscope (TESCAN vega3, Brno, Czech Republic). The samples were placed on an aluminum tape and sputter-coated with gold (Desk Sputter Coater DSR1, Nanostructural Coating Co., Isfahan, Iran) [24].

### 2.5. X-Ray Diffraction

An X-ray diffraction analysis of proteins/gum mixtures or conjugates was carried out by a Bruker ASX D8 X-ray powder diffractometer (Bruker, Billerica, MA, USA) with Cu Ka radiation operated at 40 kV and 40 mA. Analysis was performed between 5° and 70° (2θ) with 0.02° step.

### 2.6. Sodium Dodecyl Sulfate Polyacrylamide Gel Electrophoresis (SDS-PAGE)

Slab SDS-PAGE was settled based on the discontinuous buffer system of Laemmli [26]. Mixtures and conjugates were mixed with a loading buffer to create a final concentration of 1 mg/mL. Electrophoresis was performed at 25 mA and the gel was stained with Coommassie Brilliant blue R-250 (0.25%).

### 2.7. Zeta Potential

The zeta potential of mixtures and conjugates (at 0.1% *w*/*w* concentration) was measured by a Zetasizer (SZ100, Horiba, Japan).

### 2.8. Differential Scanning Calorimetry (DSC) and Thermogravimetric Analysis (TGA)

Almost 5 mg of mixtures and conjugates were analyzed by a differential scanning calorimeter and Thermogravimetric analysis (TGA) (Perkin-Elmer, Beaconsfield, UK). Analysis was performed in the temperature range of 30–230 °C under nitrogen flow and with a heating rate of 10 °C/min [27].

### 2.9. Temperature Effects on Viscosity

The effect of temperature on the viscosity of mixtures and conjugates was studied by a Rapid Visco Analyser (RVA Starch Master 2, Perten, Australia). Various dispersions (25 mL) of mixtures and conjugates were prepared at 0.3% *w*/*v* and then analyzed. The changes in viscosity were monitored by heating from 50 to 95 °C and, then by cooling from 95 to 50 °C. The paddle rotation speed was 160 rpm [28].

### 2.10. Apparent Viscosity Measurement

The effect of shear rate on the apparent viscosity of mixtures and conjugates was determined by a Brookfield viscometer (DV2 Pro II, Brookfield Engineering Laboratories, Middleboro, MA, USA) equipped with spindle cp51. The commonly used models including Power law, Bingham, and Casson were used for data fitting [28].

### 2.11. Emulsion Preparation

Protein/BSG mixtures or conjugates (0.075 g) were dispersed in double distilled water (DDW) (17.50 g) and hydrated at 25 °C for 24 h. After that, canola oil (7.50 g) was gradually added, and homogenized by Ultra-Turrax (T18, IKA, Staufen im Breisgau, Germany) at 15,000 rpm for 2 min, followed by sonication (SONOPULS, HD3200, Bandelin Co., Berlin, Germany) for 5 min at 150 W.

#### 2.11.1. Emulsion Activity

The emulsion activity index (EAI) of protein/BSG mixtures or conjugates was measured according to the literature [28]. Emulsion samples (100 mL) were diluted in DDW (25 mL) and absorption was measured at 500 nm. EAI was determined by Equation (1):

*EAI* (m^2^/g) = (2 × 2.303 × *D* × *A*)/(*I* × *Φ* × *C* × 10,000), (1)

where *A* is the absorbance at 500 nm, *I* is the optical path length of the cuvette (m), *D* is the dilution factor, *Φ* is the oil volume fraction, and *C* is the amount of emulsifier in aqueous phase (g/cm^3^).

#### 2.11.2. Emulsion Stability

Emulsions were poured into in 15 mL PE tubes, capped and stored at room temperature. The gravitational stability of emulsions was investigated after 1, 3, 7, 10, and 17 days according to the method described by Kheynoor et al. [29].

#### 2.11.3. Droplet Size

The droplet size distribution of nanoemulsions was determined by a dynamic light scattering (DLS) technique (SZ100, Horiba, Japan). At first, emulsion samples were diluted 100 times with DDW. Then, volume weighted diameter (D_4,3_) and distribution width (Span) were analyzed over time [30].

#### 2.11.4. Zeta Potential

The zeta potential of samples was evaluated over time by a DLS instrument (SZ100, Horiba, Japan). Samples were diluted 100 times with DDW before measurement. The electrophoretic mobility of the samples was transformed to zeta potential using the Smoluchowski equation [31].

### 2.12. Determination of Foaming Capacity and Foam Stability

The foaming capacity and foam stability were measured at a 0.3% (*w*/*w*) concentration based on the technique reported by Naji-Tabasi and Razavi [5] with some modifications. The dispersion (20 mL) of mixtures and conjugates (0.6% (*w*/*v*) was prepared and kept for 24 h under mixing, followed by whipping vigorously at 15,000 rpm for 2 min using a homogenizer (Ultra Turrax T18, Staufen im Breisgau, Germany).

### 2.13. Statistical Analysis

The results obtained from all tests were analyzed using one-way analysis of variance (ANOVA) at *p* < 0.05 significance. Duncan’s multiple range tests were applied by SAS^®^ software (ver. 9.1, SAS Institute Inc., Cary, NC, USA) to determine significant differences.

## 3. Results and Discussion

### 3.1. FT-IR Analysis

**Figure 1:**
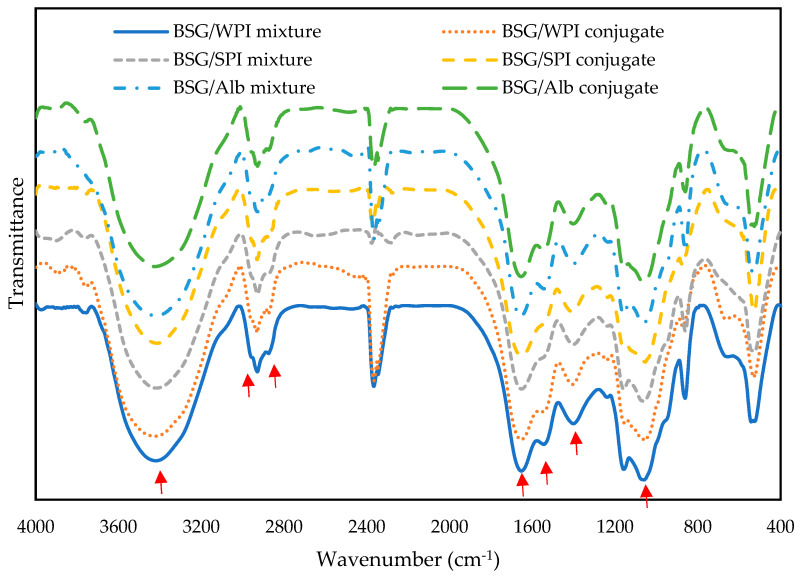
FT-IR spectra of BSG/WPI mixture, BSG/WPI conjugate, BSG/SPI mixture, BSG/SPI conjugate, BSG/Alb mixture, and BSG/Alb conjugate. Red arrows are the main peaks.

FT-IR spectroscopy is an important technique for evaluating protein–polysaccharide covalent interactions, since most characteristics regions related to both biopolymers do not overlap [32]. Figure 1 illustrates the FT-IR spectra of different protein/BSG mixtures and protein/BSG conjugates. The amide I and amide II bands, which are observed between 1650 cm^−1^ and 1540 cm^−1^, respectively, are the most specific spectral properties of proteins [33]. The main changes after conjugation were observed at around 850 to 1150 cm^−1^. The peaks at 1653.26–1655.67 and 1542.85–1545.39 cm^−1^ were attributed to amide I (C=O stretching) and amide II (N-H bending), respectively (Table 1). The band around 3420 cm^−1^ was related to the inter- and intra-molecular hydrogen bonds. This band is broad due to the overlapping of OH and -NH bands. The strong band between ~2980 and 3100 cm^−1^ is assigned to C-H stretching vibration [34]. The C=C (1653 cm^−1^) and C=N (1402 cm^−1^) stretching vibrations are the main bands in BSG. The C=O stretching was observed at 1055 cm^−1^ [35,36].

Protein/polysaccharide conjugation decreases the peak intensity of NH_2_ functional groups and increases the bands related to Amadori ingredients (C=O) and Schiff base (C=N) [37]. The fluctuations in the band intensity of C-O and C-N functional groups (at 1653 and 1545 cm^−1^, respectively) might reflect the conjugation phenomenon, and thus, the changes in the secondary structure of proteins [38]. When a protein/polysaccharide mixture is heated under certain conditions, the bands intensity at 1545 and 1055 cm^−1^ is increased [11] and a possible new band at 2870 cm^−1^ is observed. These changes are due to the formation of a Schiff base (C=N double bond) during the reaction between carbonyl groups of polysaccharides and amino groups of proteins. C-N stretching in the Schiff base appears at 1610–645 cm^−1^ depending on the ligand type [38].

### 3.2. Microstructure Properties

**Figure 2:**
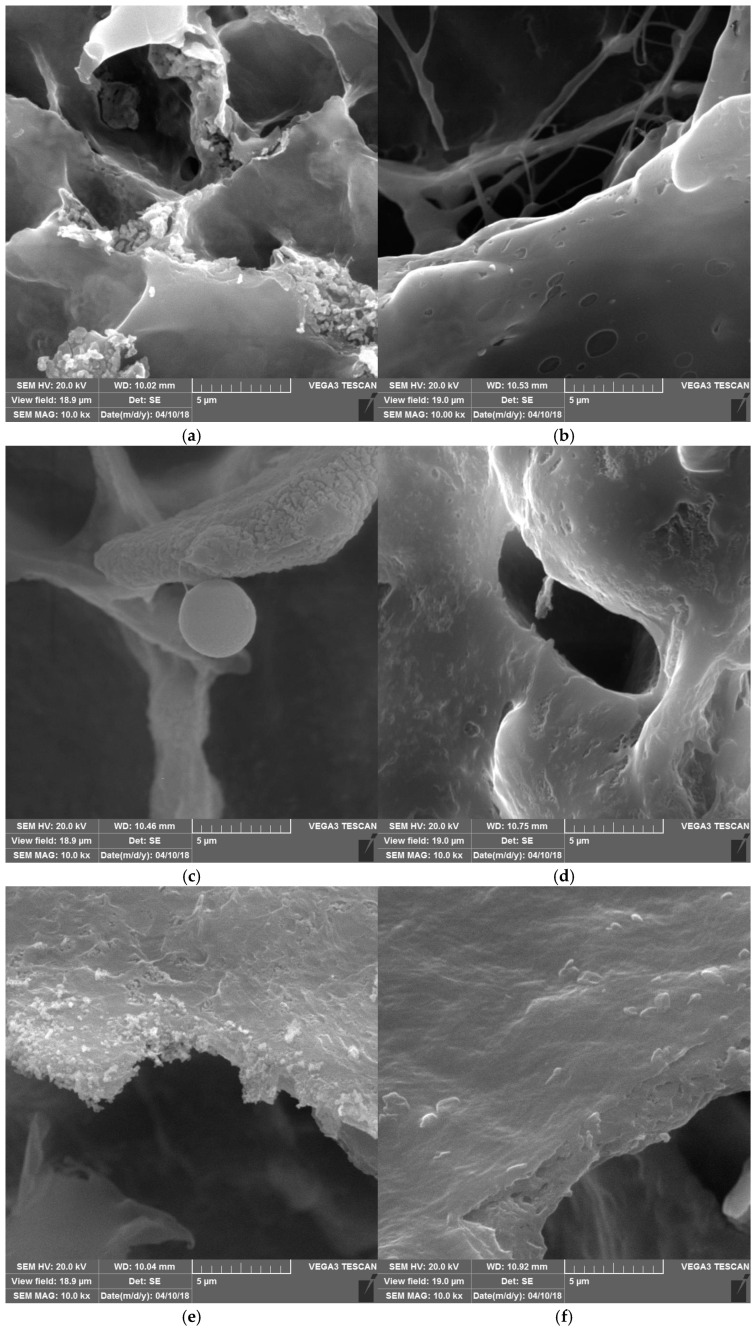
Scanning electron micrographs of (**a**) BSG/WPI mixture, (**b**) BSG/WPI conjugate, (**c**) BSG/SPI mixture, (**d**) BSG/SPI conjugate, (**e**) BSG/Alb mixture, and (**f**) BSG/Alb conjugate (10 days, T = 60 °C at pH = 8.5). Magnification 10,000×; scale bar = 5 µm.

Scanning electron microscopy is usually applied to study the microstructure of polysaccharides/proteins mixtures and conjugates [39]. Changes in the microstructure during conjugation are finally reflected in the techno-functional properties of conjugates. The SEM micrographs of mixtures and conjugates are shown in Figure 2 (or Figure S1). Aggregation or association was not observed in non-conjugated samples (Figure 2a,c,e). However, in conjugated samples (Figure 2b,d,f), the surface morphology revealed close associations and more compact microstructure. Niu et al. [40] found changes in the wheat germ protein (WGP) surface structures upon conjugation with dextran. They reported that the WGP particles were large, irregular and uneven, while WGP/dextran conjugates were thin sheets. A similar observation of conjugated microstructure was reported by Yadav et al. [41] for the mixtures and conjugates of milk protein/saccharide.

### 3.3. X-Ray Diffraction

**Figure 3:**
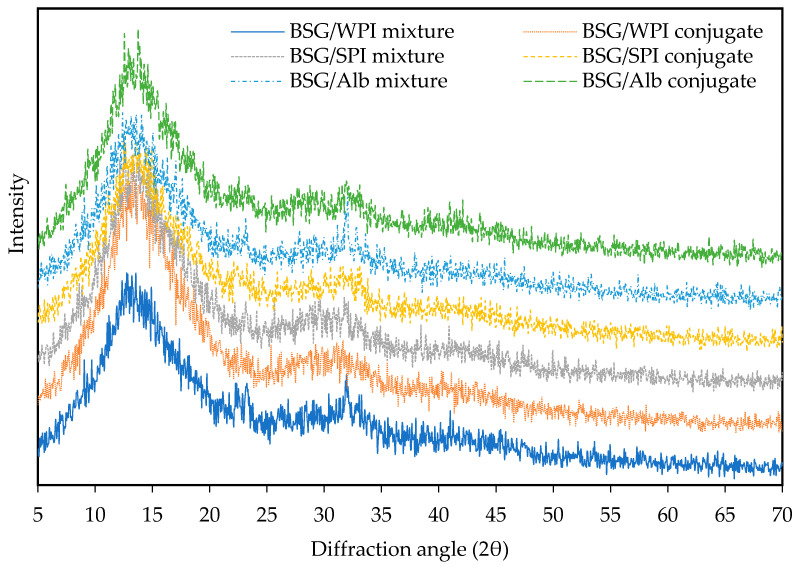
X-ray diffraction patterns of various proteins/BSG mixtures and conjugates.

X-ray diffraction was applied to evaluate the effect of conjugation on the amorphous state of biopolymers. As illustrated in Figure 3, various protein/BSG mixtures and conjugates exhibited fairly similar diffraction patterns. After mixing BSG with WPI, SPI, and Alb, the mixture showed broader peaks at 2θ values of 12.9° and 32.1°, 13.4° and 32°, as well as 13.2° and 32.1°, respectively. For the conjugates of BSG with WPI, SPI, and Alb, the position of crystalline peaks appeared at 2θ values of 13.3° and 31.7°, 13.7° and 31.8°, as well as 13.95° and 31.7°, respectively. These results showed that the conjugation of proteins with BSG does not affect the amorphous state of biopolymers. Slight changes in peak intensity were observed at 2θ around 13°. Similar results were reported in conjugation between gum Arabic and canola protein isolate [42].

### 3.4. Protein Analysis (SDS-PAGE)

**Figure 4:**
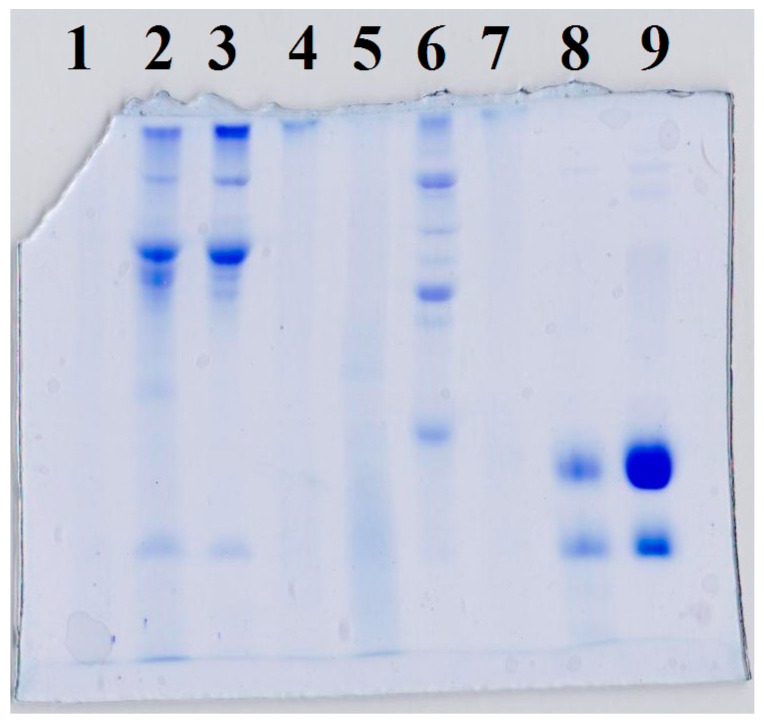
SDS-PAGE patterns of (2) BSG/WPI mixture, (3) BSG/WPI conjugate, (5) BSG/SPI mixture, (6) BSG/SPI conjugate, (8) BSG/Alb mixture, and (9) BSG/Alb conjugate. (1), (4) and (7) are hole without any sample.

The SDS-PAGE patterns of mixtures and conjugates are shown in Figure 4. The bands of BSG/WPI conjugate, BSG/SPI conjugate and BSG/Alb conjugate were sharper than those of BSG/WPI mixture, BSG/SPI mixture, and BSG/Alb mixture because of the conjugation reaction, as it caused higher molecular weight. The sharper bands observed in SDS-PAGE image probably originated from the addition of a considerable amount of BSG molecules to WPI, SPI, and Alb, and thus changes in protein conformation, disulfide-sulfhydryl bands, and interaction of protein molecules with each other [43]. The variation in band intensity was reported in many studies during the conjugation of various polysaccharides with LZM [44], milk proteins [41], and soy protein isolate [45]. Similarly, in this study, the SDS-PAGE confirmed that the conjugation of WPI, SPI, and Alb with BSG caused the formation of molecules with higher molecular weights.

### 3.5. Differential Scanning Calorimetry (DSC) and Thermogravimetric Analysis (TGA)

**Figure 5:**
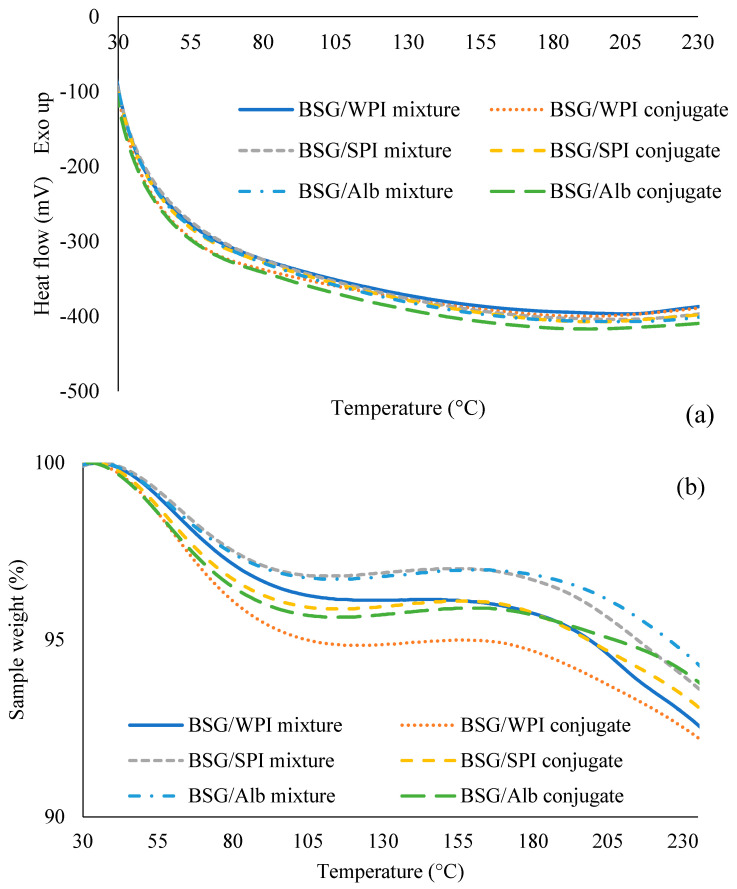
DSC thermograms (**a**) and TGA curve (**b**) of BSG/WPI mixture, BSG/WPI conjugate, BSG/SPI mixture, BSG/SPI conjugate, BSG/Alb mixture, and BSG/Alb conjugate.

Protein denaturation changes the techno-functional properties. The thermodynamic stability of proteins is usually determined by the DSC technique [46]. This technique is also applied for evaluating the thermal properties of protein–polysaccharide complexes [47]. Table 2 and Figure 5 show the thermal characteristics of BSG/protein mixtures and respective conjugates. The results showed that the BSG/WPI conjugate, BSG/SPI conjugate, and BSG/Alb conjugate had lower onset (T_onset_), peak (T_midpoint_), and endset (T_endset_) temperatures than their mixtures. However, the denaturation enthalpy (∆H) of mixtures was higher than that of conjugates. After the conjugation of WPI, SPI, and Alb with BSG, the enthalpy reduced from −52.47, −124.66 and −67.07 J/g to −203.36, −336.31 and −80.82 J/g, respectively. This reduction might be due to the aggregation and disruption of hydrophobic interactions during the exothermic reactions [48]. Protein denaturation is one of the main parameters for changing the enthalpy [47]. Takahashi et al. [49] studied the conjugation of LZM with glucose stearic acid. They reported that denaturation temperature was increased and ∆H was decreased due to the change in the α-helix in LZM and to the molecular interaction and aggregation.

Thermogravimetric analysis (TGA) showed two main steps of weight loss. The first weight loss < 90 °C was because of the evaporation of water, and the second weight loss (from 180 to 230 °C) was due to the thermal decomposition of hydrocolloids [50].

### 3.6. Rheological Results

**Figure 6:**
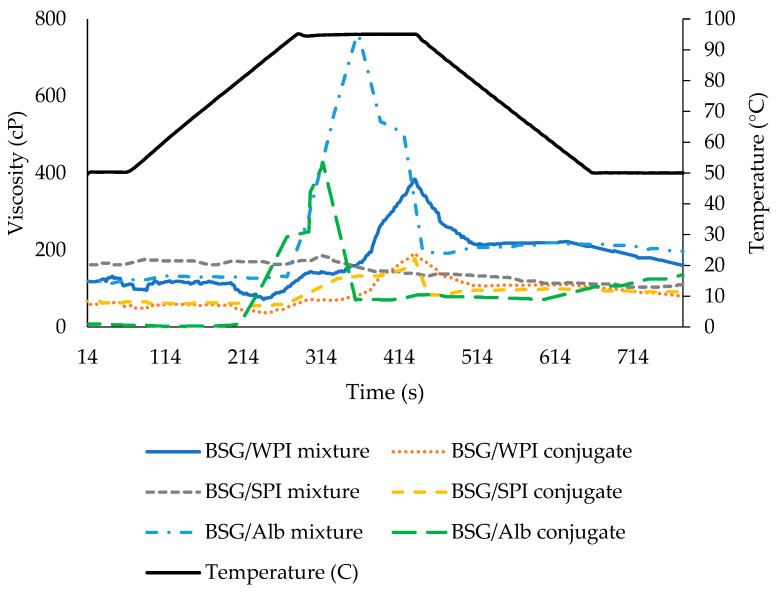
Instrumental rheological performance of BSG/WPI mixture, BSG/WPI conjugate, BSG/SPI mixture, BSG/SPI conjugate, BSG/Alb mixture, and BSG/Alb conjugate employing Rapid Visco Analyser.

The effect of temperature on the viscosity of different BSG/protein mixtures and BSG/protein conjugates (0.3% *w*/*v*) was measured by RVA. The findings are shown in Figure 6. The results indicated that in some samples, the rise in temperature to 95 °C is accompanied by increasing in viscosity, which could be attributed to the higher hydration induced by a higher temperature. The peak viscosity values of the BSG/WPI mixture, BSG/WPI conjugate, BSG/SPI mixture, BSG/SPI conjugate, BSG/Alb mixture, and BSG/Alb conjugate solution were 384, 192, 186, 178, 759, and 428 cp, respectively. Generally, the viscosity of the BSG/protein mixture was higher than that of the respective BSG/protein conjugate. This might be due to the changes in the excluded volume of biopolymers after the formation of conjugates.

**Figure 7:**
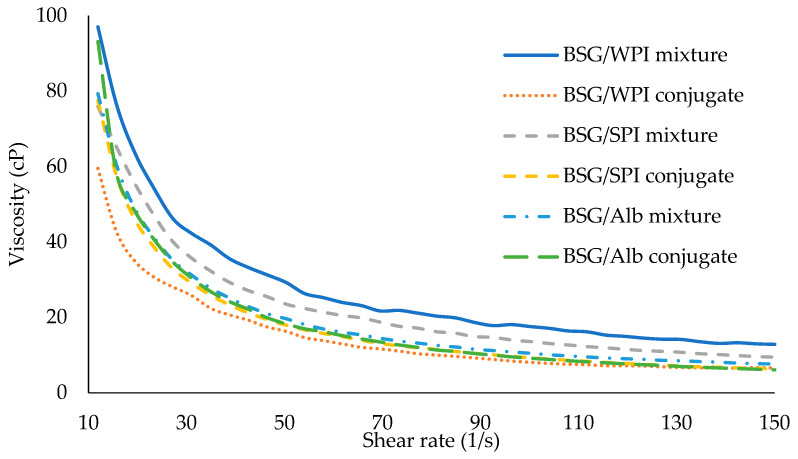
Changes in the apparent viscosity of BSG/WPI mixture, BSG/WPI conjugate, BSG/SPI mixture, BSG/SPI conjugate, BSG/Alb mixture, and BSG/Alb as a function of shear rate (0.3% *w*/*w*, 20 °C).

All samples showed shear-thinning behavior (i.e., a decrease in viscosity by increasing shear rate (Figure 7)). High MW, aggregation, and disentanglement of polymers under shear are the main reasons for shear-thinning behavior [51]. Similar results have already been reported [4]. The results of data fitting with Power Law, Bingham, and Casson models were reported in Table 3. All models exhibited high R^2^ values (0.957–0.995). The value of flow behavior index (*n*) of Power Law model (0.01–0.26) also confirmed the shear-thinning behavior (*n* < 1) of mixtures and conjugates of BSG with WPI, SPI, and Alb (Table 3). Shear-thinning hydrocolloids are utilized to change the viscosity of foods. Taking into account the recovery after removing shear, this feature is useful during some unit operations including filling and pumping [52]. The consistency coefficient (*k*) of samples ranged from 449 to 967.8 mPa s^n^. The results showed that the conjugation of BSG with WPI, SPI, and Alb did not have a significant effect on consistency coefficient (Table 3).

### 3.7. Zeta Potential

The zeta potential shows the surface charge at the distance of the slipping plane of hydrochlorides and indicates the electrostatic repulsion among charged molecules. Table 4 reports the zeta potential values of BSG/protein mixtures and BSG/protein conjugates. The BSG/WPI mixture, BSG/SPI mixture, and BSG/Alb mixture had negative zeta potential values of −39.50, −41.60, and −43.83 mV, respectively. The negative values were attributed to the pH values above proteins’ isoelectric point and negative charges along BSG backbone. Conjugation increased the absolute values of zeta potential to −45.43, −45.30, and −51.97 for BSG/WPI conjugate, BSG/SPI conjugate, and BSG/Alb conjugate, respectively. The increase in zeta potential absolute values might be attributed to the blocking of positively charged amino groups of proteins during conjugation.

### 3.8. Emulsion Properties

#### 3.8.1. Emulsifying Activity Index (EAI) and Emulsion Stability (ES)

**Figure 8:**
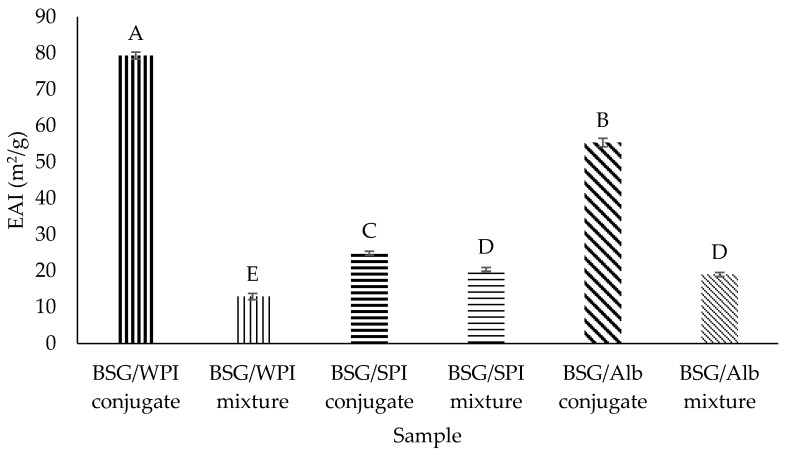
Emulsifying activity index of BSG/WPI, BSG/SPI, and BSG/Alb mixtures and respective conjugates. Different uppercase letters indicate significant differences (*p* < 0.05) between samples.

**Figure 9:**
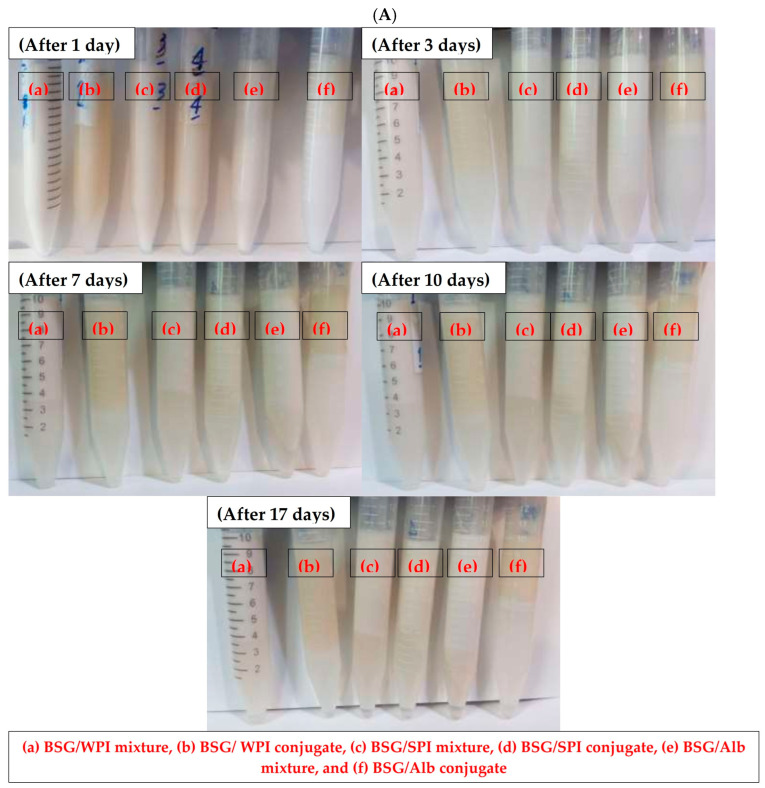

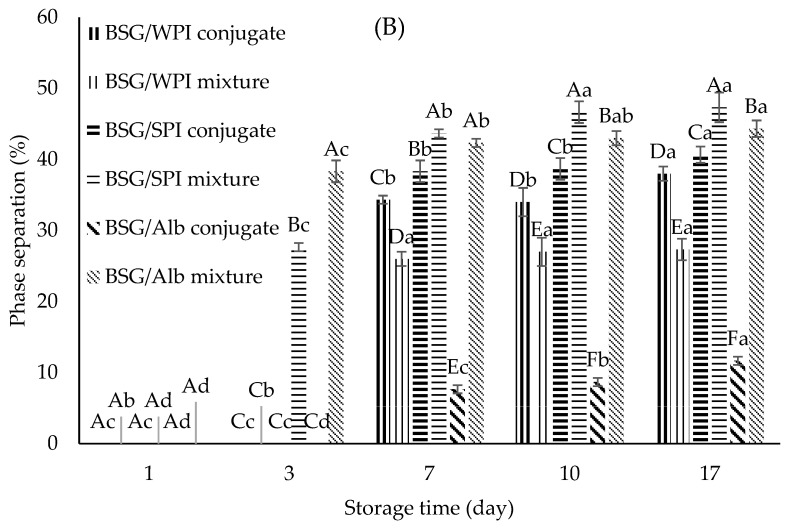
Visual observation of phase separation (**A**) and its quantification (**B**) in nanoemulsions stabilized by BSG/WPI, BSG/SPI, and BSG/Alb mixtures and conjugates. Different uppercase and lowercase letters indicate significant differences (*p* < 0.05) between samples at the same time and during the storage, respectively.

The ability of surfactants to form emulsion and to stabilize oil droplets after formation is quantified by EAI and ES, respectively [53,54]. An important techno-functional aspect of protein–polysaccharide conjugates is the improved interfacial properties induced by conjugation [55]. Generally, protein–polysaccharide conjugates have a higher ability than individual biopolymers or their mixtures to adsorb to the interface and to develop stable emulsions. Conjugation can also increase the solvation of hydrocolloids in the aqueous phase [56]. Emulsifying activity of conjugates depends on their ability to decrease the interfacial tension (IFT) between oil and water phases. IFT reduction is mediated by hydrophobic and hydrophilic moieties of conjugates toward oil and water, respectively. The EAI and turbidity of various protein/BSG mixtures and respective conjugates are shown in Figure 8. The EAIs of protein/BSG mixtures were significantly lower than those of their conjugated counterparts. In other words, the EAI in the mixture of WPI/BSG, SPI/BSG, and Alb/BSG increased from 12.94, 20.43, and 19.2 m^2^/g to 79.32, 24.89, and 55.37 m^2^/g, respectively. Polymerization during dry heat treatment plays an important role in the increase in emulsifying activity. Similarly to other emulsifiers, the adsorbed conjugates develop an interfacial film at the O/W interface, which is responsible for the emulsion stability. The type of protein had a significant effect on the ability of protein/BSG conjugates on emulsion stabilization. Emulsion samples prepared by different proteins/BSG mixtures and respective conjugates showed ES of 100% after 24 h of storage (Figure 9). After three days of storage, all proteins/BSG conjugates and the mixture of BSG with WPI revealed 100% stability. Water phase separation of around 27.7% and 38.3% was measured in the emulsion samples stabilized by SPI/BSG and Alb/BSG mixtures, respectively. Water phase separation at the bottom occurred in mixed and conjugated BSG-stabilized emulsions after 7 days of storage (Figure 9). The emulsion stabilized by the Alb/BSG conjugate revealed the lowest water phase separation. It was also homogenous, which indicated the Alb/BSG conjugate had the best emulsion stability properties. Lavaei et al. [57] studied the conjugation of gellan gum and soy protein by Maillard reaction. They reported that the conjugation increased the EAI and ES because of increasing protein solubility and improving the balance in the hydrophilic–hydrophobic character. Shen and Li [58] evaluated the functional properties of conjugated guar gum–pea protein isolate. The increase in the emulsion stability prepared by this conjugate was attributed to the formation of strong layer at the interface and thus improved steric stabilization.

#### 3.8.2. Droplet Size Distribution

**Figure 10:**
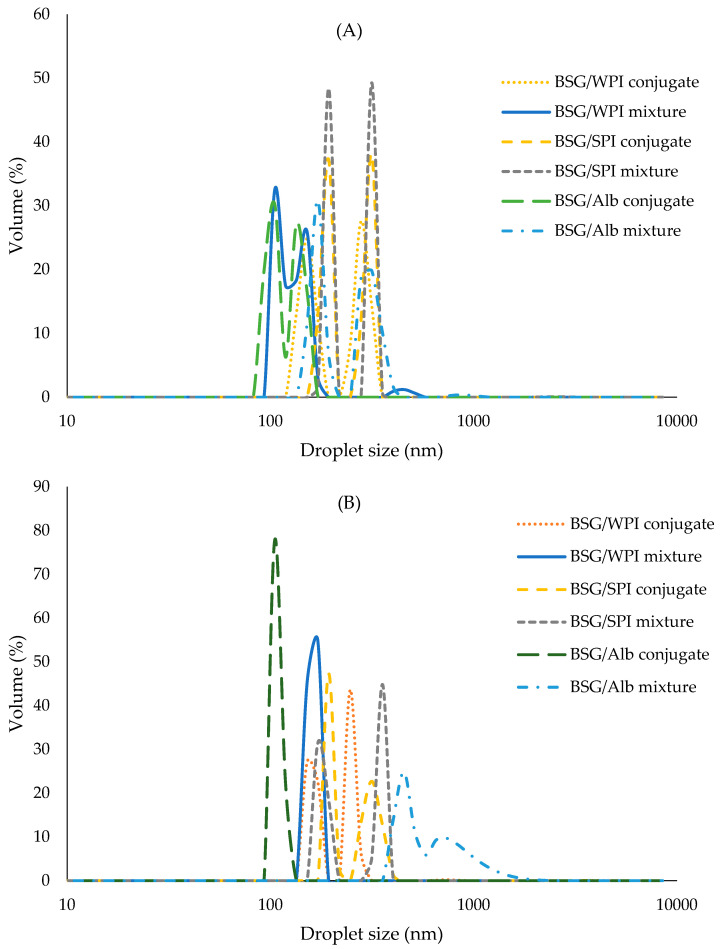

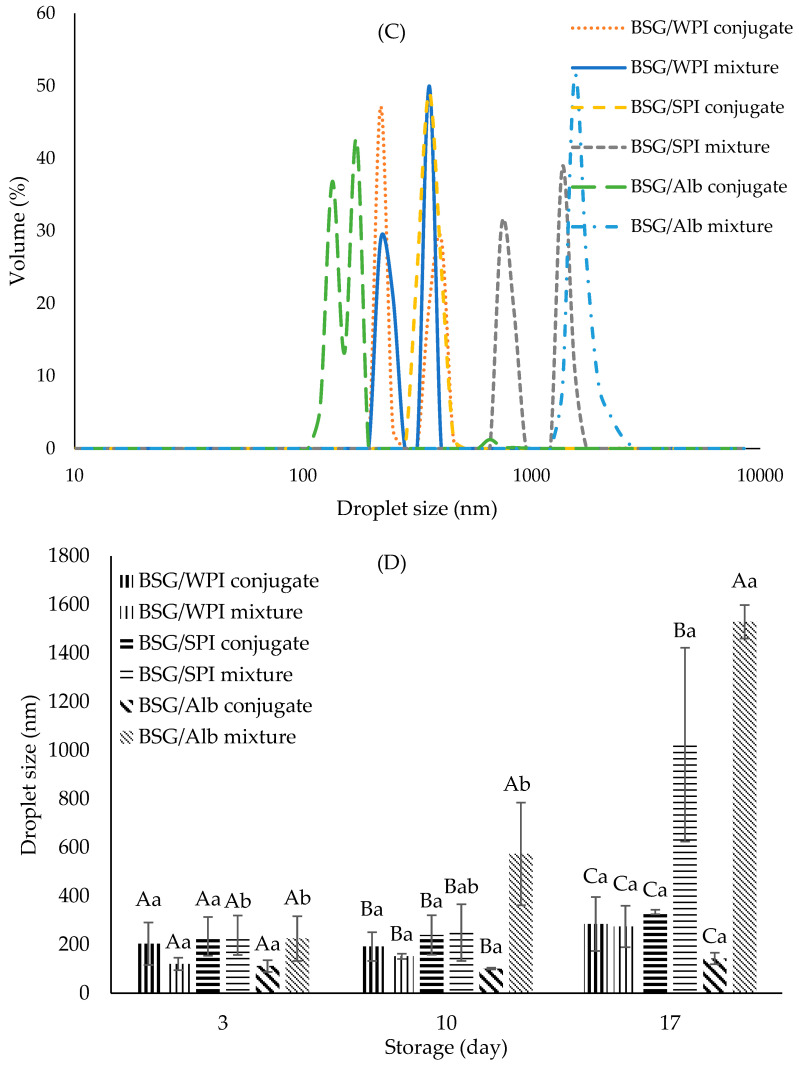
(**A**–**C**): droplet size distribution of nanoemulsions stabilized by the mixtures and/or conjugates of BSG/WPI, BSG/SPI, and BSG/Alb, respectively, during storage for 3, 10, and 17 days; (**D**) reports mean droplet diameter (nm) during storage. Different uppercase and lowercase letters indicate significant differences (*p* < 0.05) between samples at the same time and during the storage, respectively.

**Figure 11:**
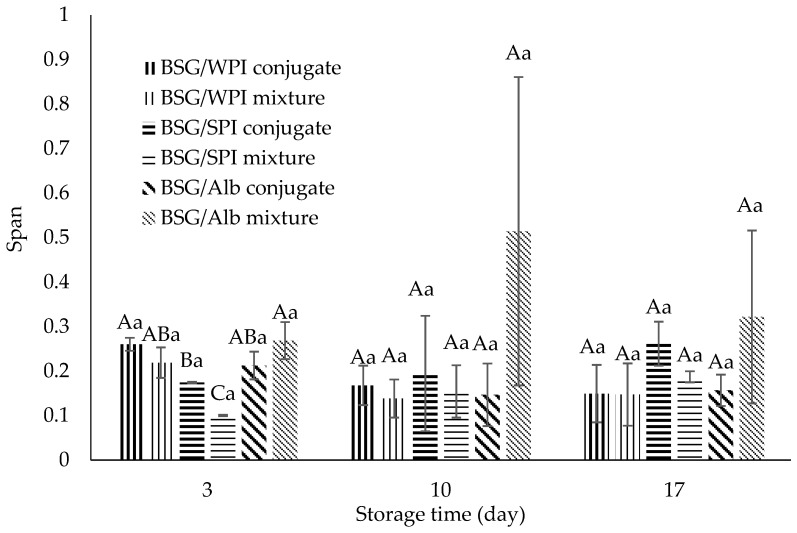
Span values of nanoemulsions stabilized by the mixtures and/or conjugates of BSG/WPI, BSG/SPI, and BSG/Alb during storage for 3, 10, and 17 days. Different uppercase and lowercase letters indicate significant differences (*p* < 0.05) between samples at the same time and during the storage, respectively.

Droplet size distribution plays an important role in the turbidity, stability, sensory experience, and rheology of food systems [59]. The volume-weighted mean droplet diameter of nanoemulsions and span (distribution width) values were measured over time and reported in Figure 10A–D and Figure 11. Protein/BSG conjugation reduced the mean diameter and span values of nanoemulsions as compared to non-conjugated protein/polysaccharide mixture. This was attributed to the significantly increasing emulsifying activity after conjugation (Figure 4). The mean droplet diameter varied from 112.1 to 239.3 nm on day 3. Nanoemulsions prepared by the mixture of SPI/BSG and Alb/BSG exhibited the largest size at day 17, while those prepared by Alb/BSG conjugates showed the smallest size during storage (112.1 and 143.3 nm at day 3 and day 17, respectively). Droplet size reduction increases the emulsions stability against gravitational separation and some other phenomena such as coalescence and flocculation. As stated by Márquez et al. [60], the droplet size is related to the emulsifier’s hydrophilic–lipophilic balance. These researchers reported that modified durian seed gum (DSG) prepared by the covalent linkage between DSG and WPI had an improved hydrophilic–hydrophobic character compared to native DSG. Akhtar and Dickinson [17] found that the conjugates of maltodextrin/WPI had high emulsifying activity and were able to prepare emulsions with smaller droplet size. Introducing hydrophobic moieties to conjugates might facilitate the adsorption rate at the interface and also lead to decreased droplet coalescence and aggregation [61,62]. The droplet size of nanoemulsions stabilized by the mixture of SPI/BSG and Alb/BSG significantly increased during storage; however, it remained relatively constant in nanoemulsions prepared by the WPI/BSG mixture and all conjugated protein/BSG counterparts. The results of span values did not show any significant difference between all samples during the storage. The span values of nanoemulsions ranged from 0.1 to 0.27 at day 3 and 0.15 to 0.32 at day 17 (Figure 11). The lower values indicated a narrower size distribution (more homogeneity).

#### 3.8.3. Zeta Potential

Table 5 reports the zeta potential values of nanoemulsions stabilized by different proteins/BSG mixtures and/or conjugates. In all samples, the O/W interface was negatively charged, arising from the negative charge along BSG backbone as well as the net negative charge of proteins at pH values above the isoelectric point. Generally, the absolute values of zeta potential decreased during storage; however, the final values were still above the minimum required values (i.e., |zeta potential| > 20 mV) for providing sufficient electrostatic repulsion among the oil droplets. Considering the high values of the zeta potential and the physical instability of nanoemulsions during storage, it can be concluded that additional factors (e.g., adsorption affinity, viscosity, interfacial film characteristics, etc.) other than the electrostatic repulsion among the dispersed droplets (i.e., zeta potential) are required for the long term stability of nanoemulsions. Chen et al. [63] evaluated the conjugation of pectin and albumin using the Maillard reaction to increase the emulsifying properties and reported that the conjugates had lower zeta potential values than the respective electrostatic complex. These researchers concluded that the involvement of more amino acid residues for the covalent linkage with carboxyl groups of pectin in the conjugates is responsible for lower values of zeta potential. Our finding is not in agreement with that of Chen, Ji, Qiu, Liu, Zhu, and Yin [63]. Except for nanoemulsions stabilized by the mixture and the conjugate of BSG/Alb, other nanoemulsions stabilized by the mixture or conjugate of same biopolymers did not significantly differ in zeta potential values after preparation. The conjugation reaction is expected to occur between the amino groups of proteins (i.e., present in the side chain of amino acid residues such as ε-amino group of lysine and/or terminal amino group of proteins) and the reducing end (i.e., carbonyl group) of polysaccharides and not carboxyl groups along backbone. Moreover, other factors such as pH, ionic strength, and microviscosity have a significant contribution to electrophoretic mobility. Similarly to our finding in the mixture and conjugate of BSG/Alb, Feng et al. [64] reported that the emulsions stabilized by the conjugates of SPI-GA had higher zeta potential absolute values than those stabilized by a non-conjugated mixture. This might be due to more blocking proteins’ amino groups after conjugation (Table 4) and hence modulating the net charge of conjugated biopolymers (i.e., the increase in contribution of negative charges). In our study, the highest physical stability was observed in the emulsions stabilized by the BSG/Alb conjugate.

### 3.9. Foaming Properties

**Figure 12:**
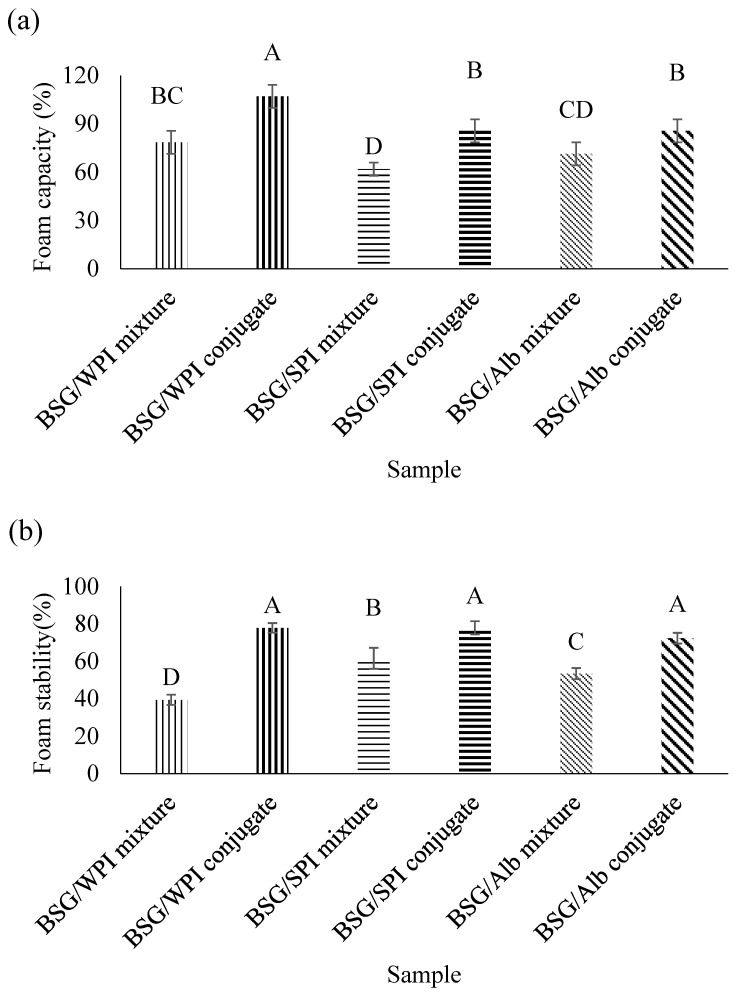
Foaming capacity (**a**) and foam stability (**b**) of BSG/WPI mixture, BSG/WPI conjugate, BSG/SPI mixture, BSG/SPI conjugate, BSG/Alb mixture, and BSG/Alb conjugate. Different uppercase letters indicate significant differences (*p* < 0.05) between samples.

Figure 12 shows the effect of conjugation on the foaming capacity of samples. The foaming capacity of WPI (78.57%), SPI (61.91%), and Alb (71.43%) in their mixtures with BSG increased to 107.14%, 85.71%, and 85.71%, respectively, after making conjugates with BSG. Therefore, conjugation increased the foaming capacity. Conjugation increases protein solubility and stability with consequent benefits for emulsification characteristics. The higher foaming capacity of conjugates compared to mixtures might be due to higher solubility, and better action as a surfactant at the air–water interface in the presence of the emulsifying and thickening effects of grafted BSG. Hamdani et al. [65] evaluated the effect of conjugation on functional properties of guar gum using egg white lysozyme. They reported that the conjugation of lysozyme improved foaming capacity (12%) and foam stability (35%). Koshani, Aminlari, Niakosari, Farahnaky, and Mesbahi [11] reported that the conjugation of tragacanthin and lysozyme enhanced foaming capacity (52.25%) and foam stability (58.23%). Also, Hashemi, Aminlari, and Moosavinasab [12] reported that the conjugation of lysozyme and xanthan gum improved foaming capacity (49.90%) and foam stability (37.80%).

Foam stability is determined as the ability of foam to maintain some of its properties during storage [66]. The gas bubbles rise to the top and may undergo deformation to form polyhedral structures [67]. In this study, the foam stability was significantly improved by conjugation with BSG (Figure 12). The foam stability of WPI (39.34%), SPI (61.57%), and Alb (53.37%) in their mixtures with BSG (non-conjugated condition) increased to 77.86%, 77.91%, and 72.32%, respectively, after formation of conjugates with BSG. The reason for better foam stabilization by BSG/protein conjugates compared to the BSG/protein mixture is likely that it improves the viscoelastic properties of interfacial film at the air–water interface in the presence of BSG. Non-grafted BSG also plays a role through increasing the viscosity of water phase. Previous studies reported that the viscosity has a significant effect on the foam stability [68]. Biopolymer networks increase the viscosity of bulk-phase and reduce the coalescence of air bubbles which increases the foam stability [67].

## 4. Conclusions

In the present research, the conjugates of WPI, SPI, and Alb with BSG were fabricated using the dry heating method via a Maillard-type reaction. The conjugates showed higher emulsifying activity and emulsion stability than non-conjugated binary mixtures of biopolymers. The BSG-albumin conjugate was the best sample. Conjugation also improved the emulsion properties. The conjugates exhibited tremendous functional properties with better foaming activities than non-conjugated samples. Furthermore, the presence of basil seed gum prevented some adverse effects of heating on proteins in powder form. Modification of natural gums can be applied for improving the techno-functional properties in food systems.

## Figures and Tables

**Table 1:** FT-IR peak assignment of BSG/WPI mixture, BSG/WPI conjugate, BSG/SPI mixture, BSG/SPI conjugate, BSG/Alb mixture, and BSG/Alb conjugate.

	Samples’ Wavenumber (cm^−1^)					
Assignment	BSG/WPI Mixture	BSG/WPI Conjugate	BSG/SPI Mixture	BSG/SPI Conjugate	BSG/Alb Mixture	BSG/Alb Conjugate
-OH stretching vibrations	3420	3424	3417	3412	3418	3425
-CH_2_- and >CH- stretching and bending vibrations	2929	2929	2928	2931	2928	2928
C-H absorption	2874	2874	2878	2873	2878	2870
C=O stretching as amide I	1653	1654	1653	1654	1655	1654
-NH twisting (amide II)	1545	1542	1540	1544	1544	1545
C=N stretching vibrations	1402	1406	1402	1408	1400	1402
-OH bending vibration	1238	1240	1238	1236	1236	1235
C-O-C stretching vibrations of glycosidic bonds	1060	1055	1060	1055	1060	1055

**Table 2:** Thermal properties of mixture and conjugate of WPI, SPI, and Alb with BSG as evaluated by DSC.

Samples	Denaturation Temperature (°C)			
	T_oneset_	Tg	T_endset_	∆H (J/g)
BSG/WPI conjugate	198.12	210.21	222.60	−52.47
BSG/WPI mixture	164.06	190.30	217.35	−203.36
BSG/SPI conjugate	182.18	207.21	226.73	−124.66
BSG/SPI mixture	162.76	192.91	222.52	−336.31
BSG/Alb conjugate	185.65	210.90	224.86	−67.07
BSG/Alb mixture	176.77	196.66	219.57	−80.82

**Table 3:** Viscosity steady shear rheological parameters of solutions (20 °C).

		Bingham			Casson			Power Law		
	Vis (cP) at 50.3 1/s	K (Pa s^n^)	τ_0 (Pa)_	R^2^	K (Pa s^n^)	τ_0 (Pa)_	R^2^	K (mPa s^n^)	n	R^2^
BSG/WPI mixture	29.27 ± 3.67 *	4.95 ± 0.72	10.24 ± 2.56	96.73 ± 1.86	1.28 ± 0.17	7.80 ± 2.28	98.73 ± 0.42	536.10 ± 185.12	0.23 ± 0.04	97.60 ± 0.56
BSG/WPI conjugate	16.30 ± 1.57	2.14 ± 0.90	7.06 ± 2.30	96.83 ± 3.50	0.69 ± 0.01	3.36 ± 1.16	98.50 ± 1.68	449.00 ± 352.26	0.26 ± 0.16	96.97 ± 3.50
BSG/SPI mixture	23.53 ± 1.83	2.96 ± 0.18	15.38 ± 8.70	95.70 ± 2.95	0.53 ± 0.09	8.64 ± 0.53	98.20 ± 1.25	646.20 ± 4.67	0.17 ± 0.02	96.67 ± 1.76
BSG/SPI conjugate	17.97 ± 2.88	0.38 ± 0.11	7.42 ± 2.83	99.13 ± 0.29	0.02 ± 0.01	7.21 ± 2.85	99.57 ± 0.15	686.97 ± 287.90	0.03 ± 0.02	99.07 ± 0.32
BSG/Alb mixture	19.64 ± 2.25	1.41 ± 0.62	9.16 ± 1.26	99.00 ± 0.40	0.07 ± 0.03	8.44 ± 1.35	99.27 ± 0.35	746.20 ± 147.77	0.08 ± 0.04	98.00 ± 0.95
BSG/Alb conjugate	18.28 ± 6.59	0.05 ± 0.05	9.43 ± 3.46	99.07 ± 0.38	0.01 ± 0.01	9.51 ± 3.61	99.50 ± 0.26	967.87 ± 390.47	0.01 ± 0.01	98.90 ± 0.56

* Data represent mean ± standard deviation of three independent repeats.

**Table 4:** Zeta potential values of BSG/protein mixtures and BSG/protein conjugates.

	BSG/WPI Mixture	BSG/WPI Conjugate	BSG/SPI Mixture	BSG/SPI Conjugate	BSG/Alb Mixture	BSG/Alb Conjugate
Zeta potential	−39.50 ± 3.54 A	−45.43 ± 2.06 B	−41.60 ± 1.05 AB	−45.30 ± 0.66 B	−43.83 ± 4.15 AB	−51.97 ± 0.81 C

Data represent mean ± standard deviation of three independent repeats. Different uppercase letters in each row indicate significant differences (*p* ˂ 0.05).

**Table 5:** Zeta potential (mV) values of nanoemulsions stabilized by the mixtures and/or conjugates of BSG/WPI, BSG/SPI, and BSG/Alb during storage for 3, 7, 10, and 17 days.

	Storage Time (day)			
Sample	3	7	10	17
BSG/WPI conjugate	−61.50 ± 0.61 Ab	−58.53 ± 2.47 Bab	−57.70 ± 0.14 BCa	−57.60 ± 1.37 CDa
BSG/WPI mixture	−60.23 ± 1.61 Aa	−58.33 ± 2.64 Ba	−58.40 ± 1.51 BCa	−57.83 ± 1.48 CDa
BSG/SPI conjugate	−61.70 ± 1.35 Ab	−57.77 ± 1.60 ABa	−56.13 ± 0.81 Ba	−56.17 ± 2.01 BCa
BSG/SPI mixture	−60.60 ± 1.45 Ab	−54.23 ± 2.47 Aa	−53.67 ± 1.00 Aa	−54.13 ± 0.68 ABa
BSG/Alb conjugate	−73.23 ± 2.97 Bc	−67.13 ± 2.25 Cb	−59.77 ± 2.03 Ca	−58.90 ± 1.57 Da
BSG/Alb mixture	−61.37 ± 1.80 Ab	−54.77 ± 0.84 ABa	−53.77 ± 0.49 Aa	−53.13 ± 0.61 Aa

Data are the average of three independent replicates ± standard deviation. Different uppercase letters in each column and lowercase ones in each row indicate significant differences (*p* < 0.05).

## Data Availability

The original contributions presented in this study are included in the article/Supplementary Materials. Further inquiries can be directed to the corresponding author.

## Supplementary Materials

The following supporting information can be downloaded at: https://www.mdpi.com/article/10.3390/foods14030390/s1, Figure S1. Scanning electron micrographs of (a) BSG/WPI mixture, (b) BSG/WPI conjugate, (c) BSG/SPI mixture, (d) BSG/SPI conjugate, (e) BSG/Alb mixture, and (f) BSG/Alb conjugate. Magnification 500×; scale bar = 100 µm.
