# Supplementary material for: Effects of Conjugation with Basil Seed Gum on Physicochemical, Functional, Foaming, and Emulsifying Properties of Albumin, Whey Protein Isolate and Soy Protein Isolate
Source: Foods. 2025 Jan 24;14(3):390. doi: 10.3390/foods14030390 (PMC11816446; doi:10.3390/foods14030390)
Supplement: Supplementary file 1 [file foods-14-00390-s001.zip › foods-3386542-supplementary.pdf]

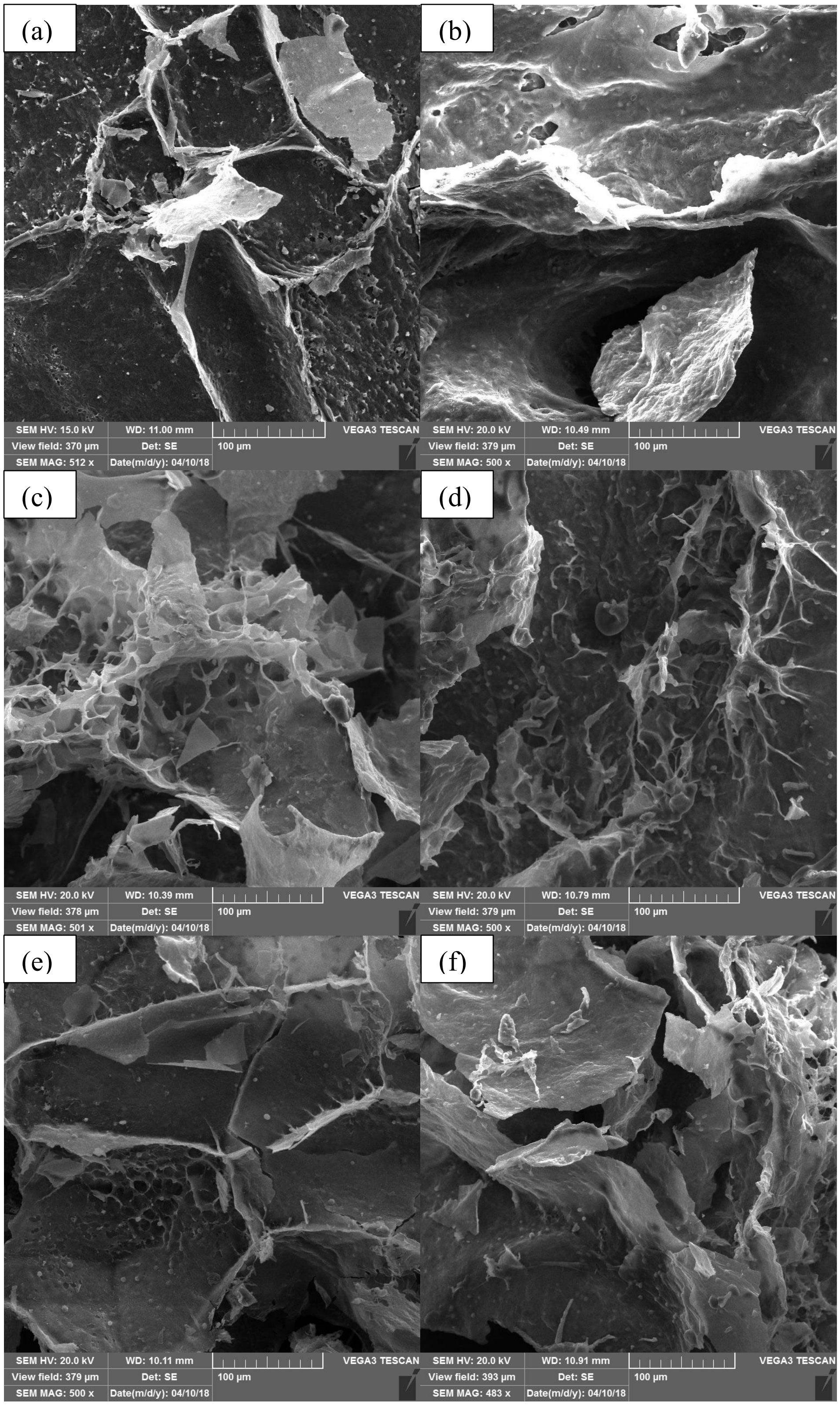
(a)
SEM HV: 15.0 kV
WD: 11.00 mm
VEGA3 TESCAN
View field: 370 µm
Det: SE
100 µm
SEM MAG: 512 x
Date(m/d/y): 04/10/18
(b)
SEM HV: 20.0 kV
WD: 10.49 mm
VEGA3 TESCAN
View field: 379 µm
Det: SE
100 µm
SEM MAG: 500 x
Date(m/d/y): 04/10/18
(c)
SEM HV: 20.0 kV
WD: 10.39 mm
VEGA3 TESCAN
View field: 378 µm
Det: SE
100 µm
SEM MAG: 501 x
Date(m/d/y): 04/10/18
(d)
SEM HV: 20.0 kV
WD: 10.79 mm
VEGA3 TESCAN
View field: 379 µm
Det: SE
100 µm
SEM MAG: 500 x
Date(m/d/y): 04/10/18
(e)
SEM HV: 20.0 kV
WD: 10.11 mm
VEGA3 TESCAN
View field: 379 µm
Det: SE
100 µm
SEM MAG: 500 x
Date(m/d/y): 04/10/18
(f)
SEM HV: 20.0 kV
WD: 10.91 mm
VEGA3 TESCAN
View field: 393 µm
Det: SE
100 µm
SEM MAG: 483 x
Date(m/d/y): 04/10/18

Fig s1. Scanning electron micrographs of (a) BSG/WPI mixture, (b) BSG/ WPI conjugate, (c) BSG/SPI mixture, (d) BSG/SPI conjugate, (e) BSG/Alb mixture, and (f) BSG/Alb conjugate. Magnification 500×; scale bar = 100 μm.
